# Supplementary material for: Mandatory Notification of Panton–Valentine Leukocidin-Positive Methicillin-Resistant Staphylococcus aureus in Saxony, Germany: Analysis of Cases from the City of Leipzig in 2019
Source: Microorganisms. 2023 May 29;11(6):1437. doi: 10.3390/microorganisms11061437 (PMC10300856; doi:10.3390/microorganisms11061437)
Supplement: Supplementary file 1 [file microorganisms-11-01437-s001.zip › S1A reporting form D.pdf]

Gesundheitsamt Stadt Leipzig
Rohrteichstraße 16-20
04347 Leipzig

**FAX: 0341 / 123 – 6905**
**E-Mail: med.einrichtungen@leipzig.de**

**Nr.**

# Ermittlungsbogen

***Nachweis von caMRSA Panton-Valentine-Leukozidin (PVL)-bildend***
*(gemäß IfSGMeldeVO vom 16.12.2012)*

---

Material: ...................... Entnahmedatum: ………….. Labor: ...................................

***Patient*in:*** Name, Vorname: ……………………….........................……
Geburtsdatum: ……………………….........................………
Wohnanschrift: ……………………….........................
……………………….........................…………
Telefonnummer ……………………….........................
E-Mail: ……………………….........................…………

☐ weiblich
☐ männlich
☐ divers

**Herkunftsland:** **Reiseanamnese:**

---

***Krankenhaus / Einrichtung / Station:*** ..............................................................

***aktuell hospitalisiert?*** ☐ **ja** **seit wann?:..................** ☐ **nein**

**zum Zeitpunkt der Befundung:** ☐ **Kolonisation** ☐ **Infektion**

**Diagnose:**
☐ **Abszess**
☐ **Furunkel**
☐ **andere**

☐ **caMRSA bekannt?** ☐ **ja** ☐ **nein**

Wenn ja, wann und wo erfolgte der Erstnachweis? ....................................................

Behandlung/Sanierung begonnen? ☐ ja ☐ nein

Wenn ja - wann und womit? ……………………………………………………………………

Kontaktpersonen aus häuslichen Umfeld ermittelt? ☐ ja ☐ nein

Datum: .............. behandelndes ärztliches Personal: .................................... Sign.: ...........
(bitte in Druckschrift ausfüllen)

caMRSA
