# Supplementary material for: Mandatory Notification of Panton–Valentine Leukocidin-Positive Methicillin-Resistant Staphylococcus aureus in Saxony, Germany: Analysis of Cases from the City of Leipzig in 2019
Source: Microorganisms. 2023 May 29;11(6):1437. doi: 10.3390/microorganisms11061437 (PMC10300856; doi:10.3390/microorganisms11061437)
Supplement: Supplementary file 1 [file microorganisms-11-01437-s001.zip › S1B reporting form EN.pdf]

Gesundheitsamt Stadt Leipzig
Rohrteichstraße 16-20
04347 Leipzig

FAX: 0341 / 123 – 6905
E-Mail: med.einrichtungen@leipzig.de

No.

Reporting form

Detection of Panton-Valentine-Leukocidin (PVL)-positive caMRSA
(according to IfSGMeldeVO from 16.12.2012)

---

material: .... isolation date: .... laboratory: ....

patient data:

surname, name: ....

date of birth: ....

address: ....

....

telephone: ....

email: ....

☐ female
☐ male
☐ diverse

country of origin: **history of travel or migration:**

---

hospital/ medical facility/ ward: ....

currently hospitalized: ☐ yes since when? .... ☐ no

at the time of diagnosis: ☐ colonization ☐ infection

diagnosis:
☐ abscess
☐ furuncle
☐ other

☐ caMRSA already known? ☐ yes ☐ no

If yes, when and where was the first detection of caMRSA? ....

Treatment/ decolonization started? ☐ yes ☐ no

If yes, when and what is used? ....

Identification of household contacts? ☐ yes ☐ no

date: .... treatment medical staff: .... signature: ....

Please fill in in block capitals

caMRSA
