# Supplementary material for: Mandatory Notification of Panton–Valentine Leukocidin-Positive Methicillin-Resistant Staphylococcus aureus in Saxony, Germany: Analysis of Cases from the City of Leipzig in 2019
Source: Microorganisms. 2023 May 29;11(6):1437. doi: 10.3390/microorganisms11061437 (PMC10300856; doi:10.3390/microorganisms11061437)
Supplement: Supplementary file 1 [file microorganisms-11-01437-s001.zip › S1C contact form D.pdf]

Stadt Leipzig

Gesundheitsamt Leipzig
Abteilung Hygiene, SG Krankenhaushygiene

Verlaufsberichtsbogen caMRSA, PVL+

PatientIn / BewohnerIn / BürgerIn

Name:<br><br>Vorname:	Geburtsdatum:<br><br>männlich ☐<br>weiblich ☐<br>divers ☐
Wohnanschrift:<br><br>Herkunftsland/Region/Reiseanamnese:<br><br>behandelnder Arzt:	

(familiäre) Kontaktpersonen

Name	Vorname	Geb. datum	Verwandschafts-verhältnis	Tel.	Anschrift

Sanierung	1. Sanierung	2. Sanierung	3. Sanierung
Datum des Sanierungszeitraumes			
Nasensalbe			
Rachenspüllösung			
Waschlotion			
orales Antibiotika			

Datum	Gesprächs-partner	Inhalte	Bemerkungen	Sig
