# Supplementary figures and images for: Mandatory Notification of Panton–Valentine Leukocidin-Positive Methicillin-Resistant Staphylococcus aureus in Saxony, Germany: Analysis of Cases from the City of Leipzig in 2019
Source: Microorganisms. 2023 May 29;11(6):1437. doi: 10.3390/microorganisms11061437 (PMC10300856; doi:10.3390/microorganisms11061437)

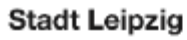[illegible]

Supplement: Supplementary file 1 [file microorganisms-11-01437-s001.zip › S1D contact form EN.pdf]
